# Impact of Type 1 Diabetes Mellitus on Skeletal Integrity and Strength in Adolescents as Assessed by HRpQCT

**DOI:** 10.1002/jbm4.10422

**Published:** 2020-11-02

**Authors:** Janani Devaraja, Richard Jacques, Margaret Paggiosi, Carolyn Clark, Paul Dimitri

**Affiliations:** ^1^ Department of Paediatric Endocrinology Sheffield Children's NHS Foundation Trust Sheffield UK; ^2^ The School of Health and Related Research, University of Sheffield Sheffield UK; ^3^ Mellanby Centre for Bone Research University of Sheffield Sheffield UK; ^4^ Directorate of Research & Innovation, Sheffield Children's NHS Foundation Trust Sheffield UK; ^5^ Sheffield Children's NHS Foundation Trust Sheffield UK

**Keywords:** DXA, OTHER, RADIOLOGY, FRACTURE RISK ASSESSMENT

## Abstract

Adults with type 1 diabetes mellitus (T1DM) are at risk of premature osteoporosis and fractures. The onset of T1DM typically starts during childhood and adolescence. Thus, the effects of DM on the skeleton may be established during this period. Studies in children with T1DM primarily use DXA with conflicting results. We present the first study in adolescents assessing the impact of T1DM on skeletal microstructure and strength using HRpQCT. We recruited 22 patients aged 12 to 16 years with T1DM who were matched by age, gender, and pubertal stage with healthy controls. Paired *t* tests were applied to assess differences in cortical and trabecular microarchitecture measurements from HRpQCT, and skeletal strength from HRpQCT‐derived microfinite element analysis. Subtotal body, lumbar, and pelvic parameters were assessed using DXA. There was no significant difference in subtotal body, lumbar spine, and pelvic BMD between T1DM and control pairs. However, tibial trabecular thickness was lower (−0.005 mm; 95% CI, −0.01 to −0.001; *p* = 0.029) and trabecular loading was lower at the distal radius (ratio of the load taken by the trabecular bone in relation to the total load at the distal end (Tb.F/TF) distal: −6.2; 95% CI, −12.4 to −0.03; *p* = 0.049), and distal and proximal tibia (Tb.F/TF distal: −5.2, 95% CI, −9.2 to −1.2; *p* = 0.013; and Tb.F/TF proximal: −5.0, 95% CI, −9.8 to −0.1; *p* = 0.047) in T1DM patients. A subanalysis of radial data of participants with duration of T1DM of at least 2 years and their matched controls demonstrated a reduced trabecular bone number (−0.15, 95% CI, −0.26 to −0.04; *p* = 0.012), increased trabecular separation (0.041 mm, 95% CI, 0.009–0.072; *p* = 0.015), an increased trabecular inhomogeneity (0.018, 95% CI, 0.003–0.034; *p* = 0.021). Regression models demonstrated a reduction in tibial stiffness (−0.877 kN/mm; *p* = 0.03) and tibial failure load (−0.044 kN; *p* = 0.03) with higher HbA1C. Thus, in adolescents with T1DM, detrimental changes are seen in tibial and radial microarchitecture and tibial and radial strength before changes in DXA occur and may result from poor diabetic control. © 2020 The Authors. *JBMR Plus* published by Wiley Periodicals LLC on behalf of American Society for Bone and Mineral Research.

## Introduction

Adults with type 1 diabetes mellitus (T1DM) have an increased risk of osteoporosis and fractures.^(^ ^1^, ^2^, ^3^ ^)^ The pooled relative risk for any fracture is 3.16;^(^ ^4^ ^)^ the risk of hip fractures is between 3.78 to 6.94 times higher than the normal adult population.^(^ ^1^, ^5^ ^)^ Previous studies have shown that adults with T1DM have reduced BMD,^(^ ^1^, ^2^, ^3^, ^6^ ^)^ and fracture risk is compounded by poor diabetic control or coexistent diabetic complications.^(^ ^3^, ^7^, ^8^ ^)^

The onset of T1DM is typically in childhood, with the peak age of onset between 10 to 14 years,^(^ ^1^ ^)^ leading to a long exposure to the effects of hyperglycemia and hypoinsulinism. Significant bone growth and remodeling occurs in childhood and adolescence with 25% of peak bone mass attained in adolescence.^(^ ^9^, ^10^ ^)^ Optimizing peak bone mass in childhood and adolescence reduces fracture risk.^(^ ^10^ ^)^ It is thus plausible that the impact of T1DM on skeletal health begins from diagnosis in childhood and adolescence, leading to skeletal changes and inadequate bone mass accrual. This may subsequently lead to an increased risk of osteoporosis and fractures in adults with T1DM.^(^ ^7^ ^)^

One of the earliest studies looking at the impact of T1DM on skeletal health in children dates back to 1948 where a loss of bone “content” was demonstrated through evaluation of conventional X‐rays.^(^ ^11^ ^)^ Since then, there have been multiple studies looking at DXA‐derived BMD in children with T1DM with conflicting results; some showing reduced BMD,^(^ ^4^, ^6^, ^10^, ^11^ ^)^ and others showing no difference in BMD compared with controls.^(^ ^12^, ^13^, ^14^ ^)^ The inconsistencies in BMD seen between studies of children with T1DM may be related to the age of the patient, time from diagnosis, and diabetic control. Despite this, studies showing no reduction in BMD have shown changes in the bone markers such as osteocalcin, PINP, and urinary pyridinoline and deoxypyridinoline, reflecting lower bone formation and an increased bone turnover.^(^ ^14^, ^15^ ^)^ Changes in bone markers can be seen within a year of onset of T1DM, suggesting that skeletal alterations may be taking place at a microarchitectural level.^(^ ^9^ ^)^ BMD may also be considered an inadequate predictor of fracture risk in DM. In a previous meta‐analysis,^(^ ^1^ ^)^ lower BMD was demonstrated in adults with T1DM, but higher BMD in adults with T2DM. Yet an increased fracture risk was observed in both conditions. Moreover, the fracture risk in T1DM is also higher than the calculated risk if it is solely based on BMD. Patients with T1DM can also still develop fractures even with a normal BMD, suggesting that fracture risk may also be driven by more subtle detrimental microstructural skeletal alterations.^(^ ^15^ ^)^

Changes in bone microarchitecture that influence bone quality and strength may precede observable changes in DXA‐derived bone mass in children. Comparable studies using pQCT in children have demonstrated detrimental changes in the cortical compartment in children and adolescents with T1DM.^(^ ^16^, ^17^ ^)^ In participants with T1DM, Bechtold and colleagues demonstrated a reduction in total and cortical cross‐sectional area in prepubertal participants and reduction in total cross‐sectional area in participants in early puberty at the radius compared with the normal reference.^(^ ^16^ ^)^ Saha and colleagues showed reduced bone cross‐sectional size, and reduced cortical BMD and cross‐sectional area in adolescents with T1DM compared with healthy controls.^(^ ^17^ ^)^ However, pQCT provides limited information about the cortical and trabecular microarchitecture, and does not provide proxies of bone strength calculated using finite element analysis. HRpQCT was therefore used in this study as it provides high‐resolution images of the cortical and trabecular microarchitecture to a resolution of 82 micrometers and estimated bone strength parameters.^(^ ^18^, ^19^ ^)^

We hypothesized that detrimental changes in bone mass and the cortical and trabecular bone microarchitecture, and proxies in bone strength will be seen in adolescents with T1DM compared with healthy controls. Thus our objectives were to determine: (i) whether T1DM causes changes in cortical and trabecular bone microarchitecture and proxies of bone strength in adolescents; (ii) if T1DM causes changes in subtotal body, lumbar spine, and pelvic bone mass; and (iii) if glycemic control and/or duration of diabetes has an impact on cortical and trabecular bone microarchitecture and proxies of bone strength in adolescents with T1DM.

## Participants and Methods

This study was approved by the South Yorkshire Research Ethics Committee. All investigations were carried out in accordance with the ethical standards laid down in the 1964 Declaration of Helsinki and its later amendments and in accordance with the International Conference on Harmonization Good Clinical Practice guidelines. All participants gave fully informed written consent prior to their participation.

Adolescents from a white population aged 12 to 16 years with T1DM were recruited from pediatric outpatient diabetes clinic at Sheffield Children's Hospital, UK. Control participants were recruited via advertisements through emails, social media, and as siblings of participants with T1DM. Recruitment took place between October 2016 and April 2018. Adolescents with an active malabsorption condition, metabolic bone disease, renal disease, immobilization of greater than 3 months, a known skeletal disorder, a fracture history within 12 months of consent, an active eating disorder, or who were on medications that can affect bone metabolism were excluded from the study. Fracture history was assessed in both groups. The medical notes of patients with T1DM were reviewed to determine age of diagnosis, duration of T1DM, and to obtain average HbA1C over the 1 year prior to the study. As the study involved exposure to radiation, female participants underwent a urine pregnancy test.

Anthropometry was undertaken with subjects wearing light clothing. Height was measured using a portable stadiometer (SECA 214 portable stadiometer, Birmingham, UK) to the nearest 1 mm and weight to the nearest 0.1 kg using electronic balance scales (SECA 770 digital weighing scales). BMI was calculated as weight (kg)/height^2^ (m^2^). Pubertal assessment was conducted using Tanner stage self‐assessment cards. Total body less head, lumbar spine, and pelvis BMD (BMD – g/cm^2^); bone mineral content (BMC – g); bone area (BA – cm^2^); and fat and lean mass (total and percentage) were measured using the Discover A densitometer (Hologic Inc., Bedford, MA, USA). Device stability was monitored using an anthropomorphic spine phantom, and weekly scans of the standard quality control (European Spine Phantom; QRM—Quality Assurance in Radiology and Medicine, Moehrendorf, Germany) were also performed using the manufacturer's software.

**Fig 1:**
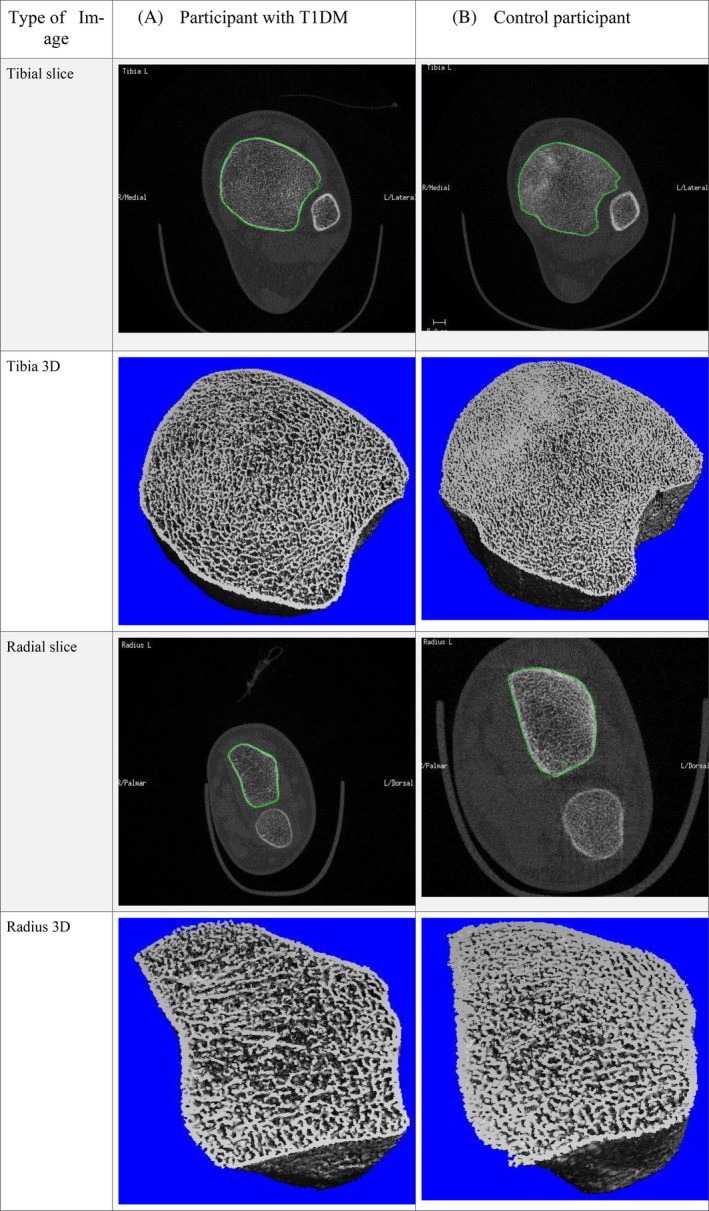
Tibial and radial images obtained from HRpQCT for a participant with type 1 diabetes mellitus (A) and an age‐, pubertal stage‐, and gender‐matched control (B).

HRpQCT was performed on the nondominant, nonfractured limb (Fig. 1). The image acquisition and analysis of a 9‐mm–defined section of the ultradistal radius and tibia scanned was performed using the standard built‐in software (XtremeCT, version 6.0, Scanco Medical AG, Brüttisellen, Switzerland). The scanning methodology has previously been described.^(^ ^18^, ^19^ ^)^ In all postpubertal participants with fused tibial and radial growth plates, a reference line was placed on the scan image at the endplate of the distal tibia and on the notch on the articular surface of the distal radius to indicate the position of the first measurement slice (22.5 and 9.5 mm proximal from the reference line for the tibia and radius, respectively). In prepubertal participants and in those participants with open tibial and radial growth plates, the reference line was placed on the scan image at the proximal end of the growth plate to indicate the position of the first measurement slice (1 mm proximal from the reference line). A single stack of parallel CT slices (110 slices = 9.02 mm) for each site was acquired in the high‐resolution mode (image matrix = 1536 × 1536). Daily measurements of the manufacturer device‐specific phantom (Scanco Medical AG) were performed to monitor the stability of the XtremeCT. Assessment of bone strength was determined using microfinite element analysis (mFEA), inbuilt software on HRpQCT.

HRpQCT densitometric measurements included total density (mg/cm^3^), trabecular density (mg/cm^3^), and cortical density (mg/cm^3^). Measures of microarchitectural properties included trabecular number (1/mm), trabecular thickness (mm), trabecular separation (mm), bone volume fraction (%), endosteal and periosteal perimeter (mm) and cortical thickness (mm). Extended cortical analysis techniques were applied to the segmented scans using specialist software provided by Scanco Medical AG (version 6) and following the approaches described by Burghardt and colleagues^(^ ^20^ ^)^, Engelke and colleagues,^(^ ^21^ ^)^ and Nishiyama and colleagues^(^ ^22^ ^)^ to assess cortical porosity (%) and mean cortical pore diameter (μm). Measures of bone strength were determined by mFEA using software developed by Scanco Medical AG (version 1.13; FE‐solver included in the image processing language). Analysis variables included bone stiffness (kN/mm), estimated ultimate failure load (kN), the ratio of the load taken by the trabecular bone in relation to the total load at the distal end (%) and proximal end (%), and average von Mises stresses in the trabecular (MPa) and the cortical (MPa) bone.

Participants with T1DM were matched to controls according to gender, age, and pubertal status. Comparisons between groups for age, anthropometry, and bone parameters (DXA, HRpQCT, and mFEA) were made using paired *t*‐tests. Paired differences in bone parameters are presented as unadjusted data and data adjusted for height and weight using mixed effects linear model with pair as random intercept. Linear regression analysis was used to determine impact of glycemic control (as assessed by average HbA1c) and duration of T1DMDM on the skeletal parameters as assessed by HRpQCT with age and gender included in the model as independent variables. Significance was determined at a *p* value of ≤0.05. The analysis was done using the R Project for Statistical Computing.^(^ ^23^ ^)^

## Results

From the adolescents contacted through the pediatric outpatient diabetic clinics, 29 adolescents with T1DM were eligible and consented to be part of the study. From these, 22 adolescents participated in the study. Thirty healthy controls were contacted and 25 adolescents participated in the study. Data from three healthy controls were excluded from the study because they could not be matched to the existing participants with T1DM (three male participants).

Twenty‐two participants with T1DM were age‐, pubertal stage‐, and gender‐matched with healthy controls. At the time of analysis, HRpQCT data could not be obtained from two participants with T1DM (because of movement artifacts); hence, analyses of these data were from 40 participants (20 matched pairs). DXA data were obtained from all participants (22 matched pairs). Demographics of the cohort are presented in Table 1. There were 13 female matched pairs (*n* = 26) in this study. Participants with T1DM were significantly heavier (8.3 kg = paired difference and 95% CI is 2.9–13.8, *p* = 0.005) and had a higher BMI (3.0 is the paired difference with 95% CI 1.0–5.1, *p* = 0.006) than controls.

**Table 1:** Comparison of Anthropometry in T1DM and Control Groups and Mean Difference (95% CI) Matched by Age, Gender, and Pubertal Stage

	T1DM		Control		Paired difference (T1DM – control)	
	Mean	SD	Mean	SD	Mean (95% CI)	*p* Value
Age (years)	13.8	1.2	13.6	1.2	0.1 (−0.03 to 0.24)	0.130
Tanner stage	3.4	0.9	2.9	1.2	0.5 (−0.1 to 1.0)	0.091
Height (cm)	160.6	9.4	159.7	10.2	0.9 (−3.9 to 5.8)	0.693
Weight (kg)	58.1	14.6	49.8	10.2	8.3 (2.9 to 13.8)	0.005
BMI	22.4	4.4	19.3	2.5	3.0 (1.0 to 5.1)	0.006

Significance is reached at *p* ≤ 0.05. T1DM = type 1 diabetes mellitus.

Eight participants with T1DM previously had fractures; six participants sustained one fracture, and two participants had two previous fractures. Ten healthy controls previously had fractures; seven participants had sustained one fracture and three participants had two fractures.

Among the participants with T1DM, the average age of onset of diagnosis was 9.25 ± 1.62 years. The duration of DM among participants ranged from 2 months (participant was 13.1 years at time of study) to 14.5 years (participant was 15.5 years at time of study). The average HbA1C was 62.4 ± 5.38 mmol (excluding one participant who did not have a HbA1C measurement as the duration of DM was <3 months).

There were no significant differences in BA, BMC, and BMD for total body less head, lumbar spine, and pelvic sites between participants with T1DM and matched controls (Table 2). After adjusting for differences in height and weight, there were no significant differences in fat and lean mass between both groups. A further subanalysis was done by excluding participants with a short T1DM duration (<2 years) and their paired healthy controls (Table 3), and there remained no difference in bone densitometry parameters.

**Table 2:** Comparison of DXA Data for Total Body Less Head, Lumbar Spine, and Pelvis in T1DM and Control Groups and Mean Difference (95% CI) Matched by Age, Gender, and Pubertal Stage

	*N*	T1DM		Control		Paired difference^a^ (T1DM – control)		Paired difference^b^ (T1DM – control)	
		Mean	SD	Mean	SD	Mean (95% CI)	*p* Value	Mean (95% CI)	*p* Value
Subtotal body area (cm^2^)	22	1570.1	203.3	1533.5	213.8	36.6 (−64.4 to 137.6)	0.459	−53.6 (−119.0 to 11.8)	0.103
Subtotal body BMC (g)	22	1328.5	235.7	1308.4	298.3	20.1 (−124.6 to 164.7)	0.776	−89.0 (−186.9 to 8.8)	0.072
Subtotal body BMD (g/cm^2^)	22	0.841	0.049	0.844	0.089	−0.002 (−0.048 to 0.043)	0.913	−0.024 (−0.062 to 0.014)	0.209
Lumbar spine area (cm^2^)	22	45.9	6.9	45.6	8.6	0.3 (−4.9 to 5.4)	0.918	−1.3 (−5.9 to 3.4)	0.579
Lumbar spine BMC (g)	22	40.7	9.8	40.7	12.2	−0.1 (−6.4 to 6.3)	0.980	−2.4 (−8.8 to 3.9)	0.438
Lumbar spine BMD (g/cm^2^)	22	0.879	0.125	0.880	0.144	−0.001 (−0.064 to 0.063)	0.983	−0.027 (−0.098 to 0.043)	0.425
Pelvic area (cm^2^)	22	189.6	28.6	192.5	39.4	−2.9 (−23.0 to 17.1)	0.763	−11.5 (−29.7 to 6.6)	0.200
Pelvic BMC (g)	22	208.6	47.1	207.4	62.4	1.2 (−29.3 to 31.8)	0.934	−17.9 (−43.7 to 8.0)	0.164
Pelvic BMD (g/cm^2^)	22	1.094	0.140	1.059	0.140	0.034 (−0.048 to 0.116)	0.393	−0.017 (−0.090 to 0.057)	0.637
Whole‐body fat (g)	22	16842.0	7360.9	12552.5	4378.8	4289.6 (1221.7–7357.5)	0.008	350.6 (−1734.1 to 2435.3)	0.729
Whole‐body lean (g)	22	41513.3	8325.9	37276.4	7729.6	4236.9 (419.2–8054.7)	0.031	−178.0 (−2279.5 to 1923.5)	0.861
Whole‐body percentage fat (%)	22	27.7	7.1	25.0	6.2	2.8 (−1.3 to 6.9)	0.175	−0.2 (−4.1 to 3.8)	0.932

Significance is reached at *p* ≤ 0.05. T1DM = type 1 diabetes mellitus.

^a^ Unadjusted analysis using paired samples *t* test.

^b^ Adjusted for height and weight using mixed effects linear model with pair as random intercept.

**Table 3:** Comparison of DXA Data for Total Body Less Head, Lumbar Spine, and Pelvis in T1DM and Control Groups and Mean Difference (95% CI) Matched by Age, Gender, and Pubertal Stage After Participants With T1DM <2 years and the Paired Controls Were Excluded

	*N*	T1DM		Control		Paired difference^a^ (T1DM – control)		Paired difference^b^ (T1DM – control)	
		Mean	SD	Mean	SD	Mean (95% CI)	*p* Value	Mean (95% CI)	*p* Value
Subtotal body Area (cm^2^)	14	1607.1	193.9	1521.9	233.5	85.2 (−58.0 to 228.4)	0.221	−57.4 (−153.5 to 38.7)	0.215
Subtotal body BMC (g)	14	1367.9	240.2	1300.9	334.5	67.0 (−151.8 to 285.8)	0.520	−112.5 (−266.3 to 41.3)	0.136
Subtotal body BMD (g/cm^2^)	14	0.846	0053	0.843	0.104	0.003 (−0.068 to 0.075)	0.919	−0.033 (−0.094 to 0.027)	0.253
Lumbar spine Area (cm^2^)	14	45.8	6.7	46.5	8.5	−0.6 (−7.1 to 5.9)	0.842	−3.9 (−9.9 to 2.2)	0.191
Lumbar spine BMC (g)	14	41.9	10.0	41.5	12.6	0.3 (−8.4 to 9.1)	0.932	−3.9 (−13.1 to 5.2)	0.365
Lumbar spine BMD (g/cm^2^)	14	0.907	0.139	0.881	0.161	0.025 (−0.073 to 0.123)	0.586	−0.017 (−0.134 to 0.099)	0.752
Pelvic area (cm^2^)	14	196.9	22.8	191.6	44.9	5.3 (−21.9 to 32.5)	0.679	−10.8 (−37.3 to 15.7)	0.390
Pelvic BMC (g)	14	220.3	47.0	207.6	70.6	12.7 (−31.5 to 57.0)	0.545	−20.7 (−60.5 to 19.2)	0.279
Pelvic BMD (g/cm^2^)	14	1.112	0.160	1.062	0.159	0.051 (−0.075 to 0.176)	0.399	−0.034 (−0.150 to 0.083)	0.538
Whole‐body fat (g)	14	18053.5	7892.9	11701.98	4528.6	6351.5 (2285.8–10417.2)	0.005	1178.3 (−2124.0 to 4480.6)	0.449
Whole‐body lean (g)	14	42833.6	9367.3	36803.3	9159.2	6030.3 (438.6– 11,622.0)	0.037	−984.0 (−4313.4 to 2345.4)	0.529
Whole‐body percentage fat (%)	14	28.6	7.2	24.0	6.9	4.6 (−1.0 to 10.2)	0.101	1.5 (−4.6 to 7.7)	0.595

Significance is reached at *p* ≤ 0.05. *N* = Number of matched pairs; T1DM = type 1 diabetes mellitus.

^a^ Unadjusted analysis using paired samples *t* test.

^b^ Adjusted for height and weight using mixed effects linear model with pair as random intercept.

HRpQCT comparisons of the radius are presented in Table 4. Participants with T1DM carried on average 6.2% less load at the distal surface of the trabecular bone (95% CI, −12.4 to −0.03; *p* = 0.049) compared with controls. However, when T1DM participants of <2 years DM duration and their paired controls were excluded in a further subanalysis (Table 5), further significant changes in the trabecular bone were found. At the radius, participants with T1DM had a reduced trabecular bone number by 0.15 (95% CI, −0.26 to −0.04; *p* = 0.012), increased trabecular separation by 0.041 mm (95% CI, 0.009–0.072; *p* = 0.015), an increased trabecular inhomogeneity by 0.018 (95% CI, 0.003–0.034; *p* = 0.021) and carried 9.7% less load at the distal surface of the trabecular bone (95% CI, −17.3 to −2.1; *p* = 0.017) compared with controls.

**Table 4:** Comparison of HRpQCT Cortical, Trabecular, and mFEA Radial Parameters and Mean Difference Between T1DM and Control Groups Matched for Age, Pubertal Stage, and Gender Calculated by Paired *t* Tests (95% CI)

Radial HRpQCT parameters	*N*	T1DM		Control		Paired difference (T1DM – control)	
		Mean	SD	Mean	SD	Mean (95% CI)	*p* Value
Total bone area (cm^3^)	20	251.7	45.6	267.3	55.9	−15.6 (−46.3 to 15.2)	0.303
Volumetric bone density (mg/cm^3^)	20	248.5	41.4	252.0	50.8	−3.6 (−37.8 to 30.7)	0.830
Cortical bone area (cm^3^)	20	27.0	12.0	24.3	12.9	2.7 (−5.7 to 11.2)	0.504
Cortical bone density (mg/cm^3^)	20	669.9	83.2	643.3	65.1	26.6 (−15.4 to 68.5)	0.201
Cortical bone thickness (mm)	20	0.424	0.196	0.396	0.241	0.029 (−0.121 to 0.178)	0.695
Cortical bone perimeter (mm)	20	64.7	6.6	66.4	7.6	−1.7 (−5.7 to 2.3)	0.394
Cortical porosity	20	0.036	0.018	0.043	0.019	−0.007 (−0.018 to 0.005)	0.248
Periosteal perimeter (mm)	20	67.1	7.6	69.1	8.7	−2.0 (−6.6 to 2.6)	0.379
Endosteal perimeter (mm)	20	60.3	7.2	63.3	8.5	−3.0 (−7.6 to 1.5)	0.182
Trabecular bone area (cm^3^)	20	213.3	45.1	230.0	52.5	−16.6 (−46.9 to 13.6)	0.265
Trabecular bone density (mg/cm^3^)	20	167.0	32.6	186.2	38.2	−19.2 (−44.4 to 6.0)	0.127
Trabecular BV/TV	19	0.140	0.027	0.157	0.032	−0.017 (−0.039 to 0.005)	0.131
Trabecular bone number (1/mm)	20	2.14	0.23	2.21	0.17	−0.07 (−0.18 to 0.04)	0.196
Trabecular thickness (mm)	20	0.065	0.009	0.070	0.012	−0.005 (−0.014 to 0.003)	0.203
Trabecular separation (mm)	20	0.409	0.057	0.387	0.041	0.022 (−0.004 to 0.048)	0.090
Trabecular inhomogeneity	20	0.159	0.029	0.151	0.023	0.009 (−0.006 to 0.023)	0.222
Stiffness (kN/mm)	20	71.3	44.5	67.2	19.9	4.2 (−20.4 to 28.7)	0.728
Estimated failure load (kN)	20	3.58	1.85	3.46	0.95	0.12 (−0.94 to 1.18)	0.815
(Tb.F/TF) distal (percentage of load carried by trabecular bone at distal surface	20	59.2	12.6	65.4	6.3	−6.2 (−12.4 to −0.03)	0.049
(Tb.F/TF) proximal (percentage of load carried by trabecular bone at proximal surface)	20	30.3	13.5	34.3	7.2	−4.0 (−10.8 to 2.7)	0.229
Trabecular Von Mises stress (MPa)	20	5.22	1.04	5.43	0.74	−0.22 (−0.92 to 0.49)	0.534
Cortical Von Mises stress (MPa)	20	8.24	0.36	8.17	0.34	0.07 (−0.14 to 0.28)	0.510

Significance is reached at *p* ≤ 0.05.

BV/TV *=* bone volume fraction; *N* = number of matched pairs; mFEA = microfinite element analysis; T1DM = type 1 diabetes mellitus; Tb.F/TF = _______.

**Table 5:** Comparison of HRpQCT Cortical, Trabecular, and mFEA Radial Parameters and Mean Difference Between T1DM and Control Groups (With T1DM Participants of DM Duration < 2 years and Paired Controls Excluded) Matched for Age, Pubertal Stage, and Gender Calculated by Paired *t* Tests (95% CI)

Radial HRpQCT parameters	*N*	T1DM		Control		Paired difference (T1DM – control)	
		Mean	SD	Mean	SD	Mean (95% CI)	*p*Value
Total bone area (cm^3^)	13	250.8	42.8	272.1	61.7	−21.4 (−59.9 to 17.1)	0.250
Total bone density (mg/cm^3^)	13	242.9	46.6	256.3	59.0	−13.3 (−64.0 to 37.3)	0.577
Cortical bone area (cm^3^)	13	29.0	12.3	26.7	15.1	2.3 (−10.7 to 15.3)	0.711
Cortical bone density (mg/cm^3^)	13	679.8	76.9	650.2	72.8	29.6 (−26.8 to 86.1)	0.275
Cortical bone thickness (mm)	13	0.452	0.198	0.445	0.280	0.008 (−0.221 to 0.236)	0.943
Cortical bone perimeter (mm)	13	65.0	6.6	66.9	8.3	−1.9 (−6.9 to 3.2)	0.432
Cortical porosity	13	0.038	0.017	0.044	0.020	−0.006 (−0.020 to 0.008)	0.353
Trabecular bone area (cm^3^)	13	211.0	42.5	232.7	57.7	−21.7 (−59.5 to 16.1)	0.236
Trabecular bone density (mg/cm^3^)	13	155.7	26.3	187.1	45.2	−31.3 (−67.3 to 4.6)	0.082
Meta trabecular density (mg/cm^3^)	13	221.7	30.0	248.4	47.2	−26.8 (−64.0 to 10.4)	0.143
Inner trabecular density (mg/cm^3^)	13	110.1	27.3	144.7	45.7	−34.6 (−71.4 to 2.2)	0.063
Meta TB/inner TB	13	2.08	0.35	1.79	0.32	0.28 (−0.04 to 0.61)	0.081
Trabecular BV/TV	12	0.131	0.023	0.159	0.038	−0.028 (−0.061 to 0.005)	0.084
Trabecular bone number (1/mm)	13	2.06	0.20	2.21	0.20	−0.15 (−0.26 to −0.04)	0.012
Trabecular thickness (mm)	13	0.063	0.008	0.070	0.014	−0.007 (−0.020 to 0.005)	0.224
Trabecular separation (mm)	13	0.427	0.055	0.386	0.049	0.041 (0.009 to 0.072)	0.015
Trabecular inhomogeneity	13	0.170	0.026	0.151	0.026	0.018 (0.003 to 0.034)	0.021
Stiffness (kN/mm)	13	60.8	13.0	69.2	24.5	−8.5 (−28.8 to 11.9)	0.383
Estimated failure load (kN)	13	3.12	0.63	3.56	1.17	−0.43 (−1.40 to 0.54)	0.353
(Tb.F/TF)dist	13	54.6	9.7	64.3	7.0	−9.7 (−17.3 to −2.1)	0.017
(Tb.F/TF)prox	13	25.5	8.6	32.9	7.8	−7.4 (−15.1 to 0.3)	0.057
Trabecular Von Mises stress (MPa)	13	4.93	0.67	5.35	0.85	−0.43 (−1.29 to 0.44)	0.302
Cortical Von Mises stress (MPa)	13	8.22	0.36	8.14	0.36	0.08 (−0.19 to 0.36)	0.533
Periosteal perimeter (mm)	13	67.0	7.3	69.8	9.6	−2.8 (−8.4 to 2.7)	0.289
Endosteal perimeter (mm)	13	60.4	7.4	64.4	9.4	−3.9 (−10.1 to 2.2)	0.186

Significance is reached at *p* ≤ 0.05. BV/TV *=* bone volume fraction; *N* = number of matched pairs; mFEA = microfinite element analysis; T1DM = type 1 diabetes mellitus; TB = Meta TB/inner TB = meta‐to‐inner trabecular density; Tb.F/TF = ratio of the load taken by the trabecular bone in relation to the total load.

HRpQCT analyses of the tibia are presented in Table 6. Participants with T1DM had a lower mean trabecular thickness (−0.005 mm; 95% CI, −0.01 to −0.001; *p* = 0.029) and had a 5.2% mean reduction in load (95% CI, −9.2 to −1.2; *p* = 0.013) on the distal surface and 5.0% decreased load (95% CI, −9.8 to −0.1; *p* = 0.047) on the proximal surface of the tibial trabecular bone compared with controls. When T1DM participants of <2 years DM duration and their paired controls were excluded in a further subanalysis (Table 7), lower trabecular thickness remained a consistent finding in children with T1DM (−0.007 mm; 95% CI, −0.013 to −0.001; *p* = 0.029) compared with controls; however, changes in load carried by the trabecular bone were no longer significant.

**Table 6:** Comparison of HRpQCT Cortical, Trabecular, and mFEA Tibial Parameters Between T1DM and Control Children Matched for Age, Pubertal Stage, and Gender Calculated by Paired *t* Tests – Mean Difference (95% CI)

Tibial HRpQCT parameters	*N*	T1DM		Control		Paired difference (T1DM – control)	
		Mean	SD	Mean	SD	Mean (95% CI)	*p* Value
Total bone area (cm^3^)	20	838.5	175.3	906.0	147.9	−67.5 (−149.0 to 14.1)	0.100
Total bone density (mg/cm^3^)	20	240.5	45.7	237.3	33.1	3.2 (−20.1 to 26.6)	0.777
Cortical bone area (cm^3^)	20	58.2	43.9	47.1	29.3	11.1 (−4.8 to 27.1)	0.160
Cortical bone density (mg/cm^3^)	20	680.2	123.2	650.5	105.7	29.7 (−9.9 to 69.4)	0.133
Cortical bone thickness (mm)	20	0.550	0.451	0.413	0.292	0.137 (−0.022 to 0.295)	0.088
Cortical bone perimeter (mm)	20	114.3	13.8	119.3	11.3	−4.9 (−10.9 to 1.1)	0.101
Cortical porosity	20	0.045	0.021	0.048	0.019	−0.003 (−0.014 to 0.007)	0.473
Periosteal perimeter (mm)	20	120.6	19.2	125.8	16.0	−5.2 (−12.5 to 2.0)	0.146
Endosteal perimeter (mm)	20	108.7	14.1	114.0	12.0	−5.4 (−11.4 to 0.6)	0.077
Trabecular bone area (cm^3^)	20	761.1	195.1	836.4	155.6	−75.3 (−161.5 to 10.8)	0.083
Trabecular bone density (mg/cm^3^)	20	184.3	24.0	197.6	24.4	−13.3 (−28.9 to 2.2)	0.089
Trabecular BV/TV	20	0.154	0.020	0.165	0.020	−0.011 (−0.024 to 0.002)	0.088
Trabecular bone number (1/mm)	20	2.21	0.30	2.18	0.39	0.04 (−0.13 to 0.20)	0.649
Trabecular thickness (mm)	20	0.070	0.009	0.075	0.009	−0.005 (−0.010 to −0.001)	0.029
Trabecular separation (mm)	20	0.391	0.062	0.386	0.060	0.005 (−0.025 to 0.035)	0.732
Trabecular inhomogeneity	20	0.157	0.03	0.164	0.037	−0.007 (−0.025 to 0.011)	0.440
Stiffness (kN/mm)	20	195.3	27.9	207.5	37.1	−12.2 (−31.2 to 6.8)	0.194
Estimated failure load (kN)	20	10.04	1.35	10.66	1.83	−0.62 (−1.55 to 0.32)	0.185
(Tb.F/TF) distal (percentage of load carried by trabecular bone at distal surface	20	72.4	12.3	77.6	8.1	−5.2 (−9.2 to −1.2)	0.013
(Tb.F/TF) proximal (percentage of load carried by trabecular bone at proximal surface)	20	54.7	14.1	59.7	9.6	−5.0 (−9.8 to −0.1)	0.047
Trabecular Von Mises stress (MPa)	20	5.60	0.58	5.73	0.57	−0.13 (−0.41 to 0.15)	0.355
Cortical Von Mises stress (MPa)	20	8.09	0.68	8.02	0.55	0.07 (−0.17 to 0.31)	0.530

Significance is reached at *p* ≤ 0.05. BV/TV *=* bone volume fraction; *N* = number of matched pairs; mFEA = microfinite element analysis; T1DM = type 1 diabetes mellitus; Tb.F/TF = ratio of the load taken by the trabecular bone in relation to the total load.

**Table 7:** Comparison of HRpQCT Cortical, Trabecular, and mFEA Tibial Parameters Between T1DM and Control Children (With Participants With T1DM of Duration <2 years and Their Pairs Controls Excluded) Matched for Age, Pubertal Stage, and Gender Calculated by Paired *t* Tests – Mean Difference (95% CI)

Tibial HRpQCT parameters	*N*	T1DM		Control		Paired difference (T1DM – control)	
		Mean	SD	Mean	SD	Mean (95% CI)	*p* Value
Total bone area (cm^3^)	13	803.3	162.1	889.4	155.1	−86.1 (−193.5 to 21.3)	0.106
Total bone density (mg/cm^3^)	13	242.9	55.9	235.2	37.2	7.7 (−28.8 to 44.2)	0.655
Cortical bone area (cm^3^)	13	67.8	49.8	52.8	30.9	15.0 (−10.3 to 40.4)	0.221
Cortical bone density (mg/cm^3^)	13	700.8	128.3	667.7	110.6	33.1 (−29.9 to 96.1)	0.275
Cortical bone thickness (mm)	13	0.648	0.500	0.486	0.312	0.180 (−0.071 to 0.431)	0.144
Cortical bone perimeter (mm)	13	111.9	13.2	117.8	12.0	−5.8 (−14.0 to 2.3)	0.145
Cortical porosity	13	0.045	0.025	0.048	0.022	−0.004 (−0.019 to 0.012)	0.635
Trabecular bone area (cm^3^)	13	719.1	185.9	816.6	162.6	−97.5 (−213.7 to 18.64)	0.092
Trabecular bone density (mg/cm^3^)	13	177.3	23.0	190.6	23.5	−13.3 (−36.5 to 9.9)	0.235
Meta trabecular density (mg/cm^3^)	13	222.2	37.6	235.5	26.7	−13.3 (−43.0 to 16.4)	0.348
Inner trabecular density (mg/cm^3^)	13	146.8	22.5	160.2	25.9	−13.4 (−35.2 to 8.4)	0.206
Meta TB/inner TB	13	1.54	0.33	1.49	0.18	0.05 (−0.13 to 0.23)	0.588
Trabecular BV/TV	13	0.148	0.019	0.159	0.020	−0.011 (−0.030 to 0.008)	0.234
Trabecular bone number (1/mm)	13	2.15	0.23	2.06	0.37	0.10 (−0.13 to 0.32)	0.365
Trabecular thickness (mm)	13	0.069	0.010	0.076	0.009	−0.007 (−0.013 to −0.001)	0.029
Trabecular separation (mm)	13	0.402	0.046	0.405	0.058	−0.003 (−0.041 to 0.034)	0.861
Trabecular inhomogeneity	13	0.163	0.028	0.177	0.037	−0.014 (−0.038 to 0.010)	0.237
Stiffness (kN/mm)	13	194.3	27.7	205.3	40.3	−10.9 (−40.6 to 18.7)	0.437
Estimated failure load (kN)	13	9.95	1.24	10.47	1.97	−0.52 (−1.96 to 0.93)	0.452
(Tb.F/TF) distal	13	70.2	11.7	75.4	8.3	−5.2 (−10.7 to 0.3)	0.062
(Tb.F/TF) proximal	13	53.2	14.0	56.8	9.5	−3.6 (−10.2 to 2.9)	0.249
Trabecular Von Mises stress (MPa)	13	5.61	0.62	5.76	0.54	−0.14 (−0.54 to 0.26)	0.446
Cortical Von Mises stress (MPa)	13	8.20	0.67	8.15	0.52	0.05 (−0.30 to 0.40)	0.766
Periosteal perimeter (mm)	13	116.5	17.4	122.8	15.9	−6.3 (−16.0 to 3.3)	0.179
Endosteal perimeter (mm)	13	106.0	13.2	112.5	12.5	−6.5 (−14.5 to 1.6)	0.104

Significance is reached at *p* ≤ 0.05. BV/TV *=* bone volume fraction; *N* = number of matched pairs; mFEA = microfinite element analysis; T1DM = type 1 diabetes mellitus; TB = Meta TB/inner TB = meta‐to‐inner trabecular density; Tb.F/TF = ratio of the load taken by the trabecular bone in relation to the total load.

Linear regression analysis was used to determine the relationship between HbA1C and duration of T1DMDM on skeletal parameters as assessed by HRpQCT. The regression models were adjusted for age and gender because this analysis was only performed for the group of T1DM patients. There was no correlation between HbA1C or duration of T1DMDM and radial skeletal parameters once adjusted for age and gender. In contrast, an increase in one unit of HbA1C was associated with a reduction in the estimated failure load by 0.044 kN (95% CI, −0.083 to −0.006; *p* = 0.035) at the distal tibia. This difference remained significant after adjusting for age and gender (−0.045 kN; 95% CI, −0.086 to −0.003; *p* = 0.039). A reduction of 0.877 kN/mm of tibial stiffness was also associated with each unit increase in HbA1C (95% CI, −1.684 to −0.07; *p* = 0.035) and again remained significant after adjusting for age and gender (−0.923 kN/mm; 95% CI, −1.783 to −0.062; *p* = 0.037). There was no correlation between duration of DM and tibial skeletal parameters once adjusted for age and gender.

## Discussion

We hypothesized that changes in bone microarchitecture and strength occur in adolescents with T1DM, and these changes precede changes in DXA‐derived bone parameters.

In our study, we compared adolescents with T1DM with age‐, sex‐, and pubertal stage‐matched controls, thus accounting for differences in physiological maturity at the same age. We did not observe differences in BA, BMC, and BMD at the total body (less head), lumbar spine, and pelvic sites. Our results thus support other studies that have shown no differences in total body and regional BMD in patients with T1DM compared with healthy controls.^(^ ^12^, ^13^, ^14^ ^)^ However, in contrast, some studies have shown a reduction in BMD.^(^ ^4^, ^8^, ^9^, ^10^ ^)^ Inconsistencies between studies using DXA are related to the age of the population and the challenges with using areal bone density to assess bone mass and fracture risk, which inherently under‐ and overestimate bone mass in smaller and taller children, respectively.

To our knowledge, this is the first study using HRpQCT to look at skeletal microarchitecture in adolescents with T1DM. HRpQCT provides high‐resolution in vivo bone biopsy, giving insight into the microarchitectural parameters and integrity of cortical and trabecular compartments.^(^ ^18^, ^19^ ^)^ Moreover, HRpQCT provides relevant information about skeletal integrity and strength, albeit at the distal appendicular skeleton. We provide evidence for reduced bone strength at the distal radius and tibia of adolescents with T1DM, demonstrating a 6.2% mean reduction in load‐bearing at the distal surface of trabecular bone in the radius and 5.2% and 5.0% mean reduction in load‐bearing on the distal and proximal surface of tibial trabecular bone respectively, compared with healthy controls. Following subanalysis in children who had T1DM for >2 years, the reduction in load‐bearing at the distal surface of tibial trabecular load in the T1DM group was no longer present. Conversely, in the same subanalysis, a reduced trabecular bone number, increased trabecular separation, and increased trabecular inhomogeneity was observed at the radius with a 9.7% reduction in the trabecular load at the distal radius. Thus, those with a duration of T1DM >2 years demonstrated an alteration in trabecular microarchitecture that could translate into a reduction in radial strength. However, given the lack of normative data on individual HRpQCT mFe parameters, it is difficult to determine whether the identified 9.7% reduction in trabecular load at the distal radius in our T1DM cohort is physiologically significant.^(^ ^24^, ^25^ ^)^ T1DM may have a more profound impact on the radius and thus, nonload‐bearing bone and this finding may in part explain the increased fracture risk observed in adults with T1DM. Moreover, radial and tibial trabecular and bone area were lower in children with T1DM, although these differences were not significant in the analysis.

Alterations in the bone microarchitecture and loading properties of the bone were thus identified in adolescents with T1DM despite no observable change in total body or regional BMD. We speculate that the consistently observed reduction in bone mass and increased fracture risk observed in adults with T1DM may be preceded and explained by more subtle skeletal microarchitectural changes in childhood. Previously observed alterations in bone turnover markers in children with T1DM, despite no significant differences in bone density, appear to support this finding, and with our data collectively suggest that the decline in skeletal quality in children with T1DM may begin in childhood.^(^ ^14^, ^15^, ^26^ ^)^

To ensure that the significant changes in bone parameters identified between the two groups were not caused by changes in body composition, we assessed the impact of whole‐body fat mass and lean mass on HRpQCT and mFEA parameters that were significantly different between the two groups to determine the effect of body composition on these differences (data not included). We analyzed the effect of an increase per 100 grams of fat and lean mass and an increase in 1% fat mass and lean mass on each of the parameters for the diabetic group, and then adjusted the data for age and sex. Moreover, we performed a sensitivity analysis to ensure outliers did not skew the results. We found that in children with T1DM, whole‐body fat mass, percentage fat mass, and percentage lean mass were positively correlated with trabecular thickness at the radius following adjustment for age and sex. Further, an increase in percentage fat mass was also correlated with an increase in trabecular load at the distal radius. Body composition was not correlated with the relevant bone parameters at the tibia. In addition, as there was no difference in fat and lean mass observed between our cohorts, we surmise that the differences in bone parameters observed between the two groups are unlikely to be caused by changes in body composition in patients with T1DM.

In our study, at least at the tibia, a reduction in bone strength may be related to glycemic control. This concurs with other studies showing the negative impact of poor glycemic control on bone.^(^ ^8^, ^12^, ^27^, ^28^, ^29^ ^)^ Our initial logistic regression analysis suggested that the duration of DM may be related to changes in tibial microarchitectural properties; however, this relationship disappeared after adjusting for age and gender. This is in agreement with other studies.^(^ ^12^, ^29^ ^)^

In adult patients with T1DM,^(27^ ^)^ no differences in HRpQCT parameters were identified between T1DM patients without the presence of microvascular disease and healthy controls. However, T1DM patients with established microvascular changes showed lower total, trabecular, and cortical volumetric BMD, thinner radial cortex, and lower total and trabecular vBMD at the tibia.^(^ ^27^ ^)^ Despite this, no differences were observed in bone strength. Differences in the microarchitectural findings between adolescents and adults with T1DM, may in part relate to the impact of T1DM during skeletal development. Adolescence is a period of significant bone mass accrual and increased bone strength.^(^ ^30^ ^)^ Thus, the impact of T1DM may differ during phases of skeletal development and maturity.

Multiple mechanisms by which T1DM may cause impaired bone turnover and mineralization have been proposed.^(^ ^31^ ^)^ Our study showed that T1DM had a negative impact on the trabecular compartment at the radius and tibia, with others showing deterioration in the cortical bone compartment.^(^ ^16^, ^17^, ^27^ ^)^ Insulin has an anabolic effect on bone by stimulating osteoblast differentiation in the bone marrow; thus, reduction in insulin may impair bone formation at a critical time of peak bone mass accrual.^(^ ^32^, ^33^ ^)^ Adolescents with T1DM have lower osteocalcin and insulin‐like growth factor 1 levels, factors that are important in skeletal development.^(^ ^34^ ^)^ Production of advanced end glycosylation products secondary to chronic hyperglycemia have also been implicated in causing impaired bone formation.^(^ ^35^, ^36^ ^)^ T1DM also may also impair osteocyte function through its impact on sclerostin expression.^(^ ^32^, ^36^, ^37^ ^)^

Adolescents with T1DM were heavier and had a higher BMI compared with the healthy controls in our study. This corresponds with data from the National Pediatrics Diabetes Audit^(^ ^38^ ^)^ showing a trend for higher BMI in children with T1DM. It could be postulated that the higher weight and BMI has led to skeletal microarchitectural deterioration rather than T1DM, as obesity has been associated with an increased risk of fractures and change in skeletal microarchitecture in children.^(^ ^39^, ^40^ ^)^ However, the mean BMI in our T1DM cohort was within the normal range for age. Although obesity has been recognized as a risk factor for fractures, increased fat mass that is not excessive could have a positive impact on skeletal development.^(^ ^40^ ^)^ There was also no significant difference in fat and lean mass or percentage body fat between T1DM participants and paired controls in our group after adjustment for height and weight, and we further showed that the differences in trabecular parameters between our groups could not be explained by differences in fat or lean mass. Therefore, the deterioration in bone microarchitecture seen in our adolescents with T1DM cannot be explained by the higher BMI alone.

There were several limitations to our study. This was a small pilot study; thus, our ability to detect other differences in skeletal microarchitecture and integrity between the groups was limited. Studying a larger population of adolescents and children with T1DM using HRpQCT could further define the relationship between skeletal microarchitecture and duration of DM, age of onset, and glycemic control. Although we detected no significant differences in fat and lean mass between both groups and we also corrected for height and weight to remove these measures as confounding factors, matching for BMI in addition to age, gender, and pubertal stage would potentially preclude the impact of body composition skeletal microarchitecture. We recognize that the measures of bone strength using finite element analysis represent proxies of bone strength and cannot replace *ex‐vivo* bone strength analysis. However, FEA is a well‐recognized process in engineering to assess material properties; our results thus provide insight into the potential impact of T1DM on skeletal strength. The rate of fractures in our population is higher than reported in previous studies^(^ ^41^ ^)^; this may have resulted from an unconscious bias in families of participants who volunteered to be part of the study. Finally, as HRpQCT measures bone strength and microstructure of the ultradistal radius and tibia, we are unable to confirm whether the alterations observed in adolescents with T1DM reflect changes in other parts of the appendicular and axial skeleton. However, accounting for the limitations, we have found significant detrimental changes in the trabecular microarchitecture and bone strength proxies in adolescents with T1DM. Thus, we affirm our hypothesis that detrimental changes in bone microarchitecture and proxies in bone strength are seen in adolescents with T1DM, despite no significant changes in DXA‐derived bone mass.

## Conclusion

T1DM is associated with a reduction in the trabecular thickness in the tibia, and alterations in the loading properties at the ultradistal radius and tibia in adolescents with T1DM, despite there being no significant reduction in BMD. Alterations in radial trabecular microarchitecture were seen in participants who have had T1DM for at least 2 years, with no corresponding changes in BMD. Poor glycemic control was associated with a reduction in bone strength. Thus, in earlier life, bone microarchitecture and strength, rather than bone density, may better explain the increased risk of fracture observed in adults with T1DM.

## Disclosures

None of the authors have a conflict of interest.

## AUTHOR CONTRIBUTIONS

**Richard Jacques:** Formal analysis; resources; software; validation. **Margaret Paggiosi:** Data curation; investigation; resources; software. **Carolyn Clark:** Investigation; project administration. **Paul Dimitri:** Conceptualization; formal analysis; funding acquisition; methodology; project administration; resources; supervision; validation; writing‐original draft; writing‐review and editing.

### Peer Review

The peer review history for this article is available at https://publons.com/publon/10.1002/jbm4.10422.
